# Low rate of secondary interventions for post-traumatic osteoarthritis and satisfactory mid-to-long-term outcomes following tibial plateau fractures

**DOI:** 10.1186/s12891-025-08685-x

**Published:** 2025-04-30

**Authors:** Tobias Resch, Frederik Hartz, Lea Faber, Philipp Zehnder, Gregor Römmermann, Ahmed Ellafi, Peter Biberthaler, Frederik Greve

**Affiliations:** 1 Department of Trauma Surgery, TUM Universitätsklinikum, Klinikum rechts der Isar, Technical University of Munich, Ismaninger Str. 22, 81675 Munich, Germany; 2 Department of Sports Orthopaedics, TUM Universitätsklinikum, Klinikum rechts der Isar, Technical University of Munich, Ismaninger Str. 22, 81675 Munich, Germany

**Keywords:** Tibial plateau fracture, Clinical outcomes, Post-traumatic osteoarthritis, Total knee arthroplasty, Secondary procedure, Follow-up intervention

## Abstract

**Background:**

The purpose of this study was to quantify the incidence of total knee arthroplasty (TKA) and other osteoarthritis-related procedures following surgical and conservative treatment of tibial plateau fractures (TPF). Secondary goal was to analyse the long-term clinical outcomes and identify risk factors for secondary interventions and poor outcomes.

**Methods:**

All patients diagnosed with TPF at a single level 1 university trauma centre between January 1, 2008 and December 31, 2016 were retrospectively reviewed. Clinical outcomes were measured by use of the Knee injury and Osteoarthritis Outcome Score (KOOS), the International Knee Documentation Committee Score (IKDC) and the Tegner Activity Score (TAS). Joint-preserving interventions and conversions to TKA were recorded as well as demographic data, injury mechanisms, treatment specifics and complications.

**Results:**

105 cases of TPF, 89 with surgical and 16 with conservative treatment, with a median follow-up of 10.4 years (interquartile range, IQR 9–13), were included. The conversion rate to TKA was 2%, with all cases occurring in the conservative treatment group. 9% underwent a joint-preserving intervention. Higher body mass index (BMI) was associated with an increased risk for secondary intervention (HR 1.4, *p* = 0.03). The overall KOOS was 78.7 (IQR 69–87) for surgical and 86 (IQR 70–93) for conservative treatment. The IKDC score was 63.6 ± 16.5 for surgical and 66.3 ± 22.2 for conservative treatment and the median TAS was 3 (IQR 3–4 vs. 3–6) for both groups. In the surgical treatment cohort, a negative correlation was found between Schatzker classification (Spearman´s r_p_ = -0.24, *p* = 0.03), duration of surgery (Spearman´s r_p_ = -0.23, *p* = 0.03), American Society of Anesthesiologists (ASA) risk classification (Spearman´s r_p_ = -0.28, *p* = 0.01) and the IKDC score. A higher TAS was observed for non-smokers (median 3, IQR 3–4) compared to smokers (median 2.5, IQR 2–3, *p* = 0.02).

**Conclusions:**

There was a low incidence of TKA and joint-preserving, osteoarthritis-related procedures following TPF. Both conservative and surgical treatments can achieve satisfactory long-term clinical outcomes, when appropriately indicated. Obese patients are at increased risk for secondary interventions. The expectations of patients with a higher ASA risk score and complex fractures, accompanied by longer surgical times, should be managed carefully to ensure a realistic outlook on functional outcomes.

## Background

Tibial plateau fractures (TPF) are severe intra-articular injuries with high complication risk and functional impairment [1]. Most of these fractures are treated surgically, with exceptions made for undisplaced, stable fractures or patients with serious co-morbidities that pose high perioperative mortality risk. The primary goals of surgical treatment for TPF are to restore the anatomical joint surface and lower limb alignment, as well as to achieve stable fixation that facilitates early functional rehabilitation [2]. Open reduction and internal fixation (ORIF) is a well-established treatment option for complex TPF. Various surgical approaches allow an individualized procedure depending on the fracture pattern, while anatomically preformed locking plates ensure stable fixation. External fixators are used for temporary stabilization in staged procedures and serve as a minimally invasive definitive therapy of fractures with compromised soft tissue integrity. Arthroscopically assisted reduction and internal fixation (ARIF) allows for accurate diagnosis and management of intra-articular lesions, as well as precise reduction control in less displaced fractures [2, 3].

Post-traumatic osteoarthritis (PTOA) is a common and serious complication following surgical treatment of TPF, in addition to infection, mal-union/non-union and knee stiffness [4]. Factors contributing to the development of PTOA include the severity of traumatic cartilage damage, meniscal lesions, post-traumatic knee laxity due to ligament injuries, malalignment, and persistent joint incongruities [5]. Total knee arthroplasty (TKA) is the treatment of choice for end stage osteoarthritis of the knee after failed conservative management, although outcomes for PTOA are worse than TKA for primary arthritis [6]. A recent systematic review reported a conversion rate to TKA of 5% following TPF [7]. This review found higher conversion rates in studies with more than five years of follow-up compared to those with shorter follow-up durations. This suggests that the rate continues to increase over time, highlighting the need for investigations with long-term follow-up. Additionally, many included studies consist of small cohorts and the outcomes of conservatively treated patients are reported infrequently. The existing literature with large patient populations is primarily based on analyses of administrative data bases, which often provide limited information regarding patient characteristics, injury mechanisms, fracture types, treatment specifics and functional outcomes [8–13]. Consequently, these register studies are unsuitable for determining prognostic factors for conversion to TKA and poor outcomes. Joint-preserving follow-up procedures after TPF such as intra-articular injections or arthroscopic interventions are also rarely documented [11–14]. Furthermore, patients often underestimate the severity of their injury and hold high expectations for treatment outcomes, highlighting the need for more research on long-term clinical outcomes to enhance patient education and foster realistic expectations [15].

Therefore, the objectives of this study were to: (1) quantify the incidence of TKA and other secondary procedures following TPF, (2) analyse the long-term clinical outcomes of TPF and (3) identify risk factors associated with secondary interventions and unfavourable outcomes after TPF.

## Materials and methods

### Patients

This retrospective cohort study was approved by the ethical committee of the Technical University of Munich (No: 2023-14-S-NP) and conducted in accordance with the ethical standards of the 1964 Declaration of Helsinki and its later amendments. All patients treated for TPF at a single level I university trauma centre between January 1, 2008 and December 31, 2016 were reviewed for enrolment. Patients were recruited via invitation letters and informed consent was obtained from each patient. Exclusion criteria included: age < 18 years, insufficient German language skills, residence outside of Germany, missing or incorrect contact information, refusal to participate in the study, death during follow-up, mental illness with cognitive impairment, bilateral TPF, pathologic fracture, concomitant fracture of the same leg and additional injury to the ipsilateral knee during follow-up.

### Fracture treatment

Standard imaging for TPF included conventional radiographs and computed tomography (CT). Magnetic resonance imaging (MRI) was not routinely performed, its use depended on availability and the surgeon’s preference. Conservative treatment was chosen for undisplaced fractures, patients with significant co-morbidities, or those with low functional demand. This approach involved bracing, non-weight bearing for six weeks and follow-up radiographs. Surgical treatment was planned according to the fracture pattern, soft-tissue condition and patient factors. Temporary stabilization using external fixators was performed in open fractures, severe soft-tissue damage (e.g. compartment syndrome), vascular or nerve injury, polytrauma and severely displaced fractures. Open fractures were managed with debridement, lavage, systemic antibiotic therapy, negative pressure wound therapy (NPWT) and, if necessary, plastic reconstruction. Less displaced unicondylar fractures were treated with screw fixation. ORIF with locking plates (3.5 mm LCP, DePuy Synthes, Solothurn, Switzerland) was performed by use of the anterolateral, posterolateral, posteromedial and anteromedial approaches. For comminuted bicondylar fractures, two-column fixation with double plating (3.5 mm one-third tubular plate/LCP, DePuy Synthes, Solothurn, Switzerland) was often necessary and performed in a staged procedure to prevent soft-tissue complications. Subarticular defects were managed using allogenic (DIZG, Berlin, Germany) or synthetic bone grafts (ChronOs, Arthrex, Naples, USA). In simple fractures with good soft tissue conditions, ARIF was performed. Postoperatively, weight-bearing was restricted to 15 kg for six weeks followed by gradual increase of loading. Both conservatively and surgically treated patients were provided with a brace, allowing 30 degrees of flexion for 2 weeks, 60 degrees of flexion for the following 2 weeks, and 90 degrees of flexion for an additional 2 weeks. Osteosynthesis material was removed upon confirmation of bone union, if local symptoms were present, or at the patients’ request.

### Outcome measures

Patients included in this study were assessed using the German versions of the Knee injury and Osteoarthritis Outcome Score (KOOS), the International Knee Documentation Committee Score (IKDC) and the Tegner Activity Score (TAS). The KOOS has recently been validated for tibial plateau fractures [16]. The score was calculated for each subscale and overall and results were categorized into four grades as described in the literature: “excellent” (100 − 95), “good” (94 − 84), “fair” (83 − 65) and “poor” (< 65) [14]. The IKDC Score is a valid measurement tool for a wide range of knee joint diseases and injuries, ranging from 0 to 100. A score of 100 was defined as unrestricted functionality in daily life and sports without symptoms [17]. The TAS is a standardized scale of work and sports activities with eleven different activity levels to choose from. Scores between 6 and 10 require regular engagement in recreational or competitive sports [17].

In addition to the aforementioned outcome measures, a standardized questionnaire was utilized to document follow-up interventions. Joint-preserving procedures, including intra-articular injections (hyaluronic acid, corticosteroids, platelet-rich plasma), arthroscopic debridement or cartilage treatment (microfracture/microdrilling, matrix-augmented bone marrow stimulation, osteochondral transplantation, autologous chondrocyte transplantation, minced cartilage technique) and osteotomies were recorded along with conversions to TKA. If multiple intra-articular injections were administered, the timing of the first injection was noted. Demographic data, injury mechanisms, treatment specifics and complications were extracted from medical records. Trauma mechanisms were categorized into low-energy trauma (e.g. falls from a small height) and high-energy trauma (e.g. falls from a great height, bicycle accidents, skiing or other sport-related accidents, motorcycle accidents and other traffic collisions). Fractures were classified according to the Schatzker classification by reviewing radiological records.

### Statistical analysis

Statistical analysis was performed using SPSS Statistics (Version 29.0, IBM Corp., Armonk, USA). Categorical variables were described as absolute numbers and percentages. Continuous, normally distributed variables were presented as mean values with standard deviation (SD), while ordinally scaled variables and variables with a skewed distribution were described using median and interquartile range (IQR). The normality of data distribution was assessed both graphically and using the Kolmogorov-Smirnov test. Kaplan-Meier survival analysis was employed to evaluate the survival of TPF with conversion to TKA and joint-preserving procedures as endpoints. Risk factors for conversion to TKA and minor follow-up interventions in the surgically treated cohort were assessed using a Cox proportional hazards model. Factors analyzed included sex, age, body mass index (BMI), American Society of Anesthesiologists (ASA) risk classification, smoking status, fracture severity according to the Schatzker classification, time between trauma and surgery and duration of surgery. Adjusted hazard ratios (HRs) and 95% confidence intervals (CI) were calculated for each risk factor. Due to the limited sample size of the conservatively treated cohort, Cox proportional hazards modelling was not feasible for these patients. Potential correlations between risk factors and functional outcomes (overall KOOS, IKDC score and TAS) were examined using either the Pearson correlation coefficient or the Spearman´s rank correlation coefficient. Functional scores were further compared across subgroups - male vs. female gender, non-smokers vs. smokers, follow-up intervention vs. no follow-up intervention and high- vs. low-energy trauma - using the Mann-Whitney U test or t-test for independent samples. *P*-values ˂0.05 were considered statistically significant. Given the marked disparity in group sizes and fracture types, direct comparison between surgically and conservatively treated patients were deemed inappropriate. Results are therefore presented separately. Functional scores were excluded from analysis for patients who underwent TKA during the follow-up period.

## Results

**Fig. 1:**
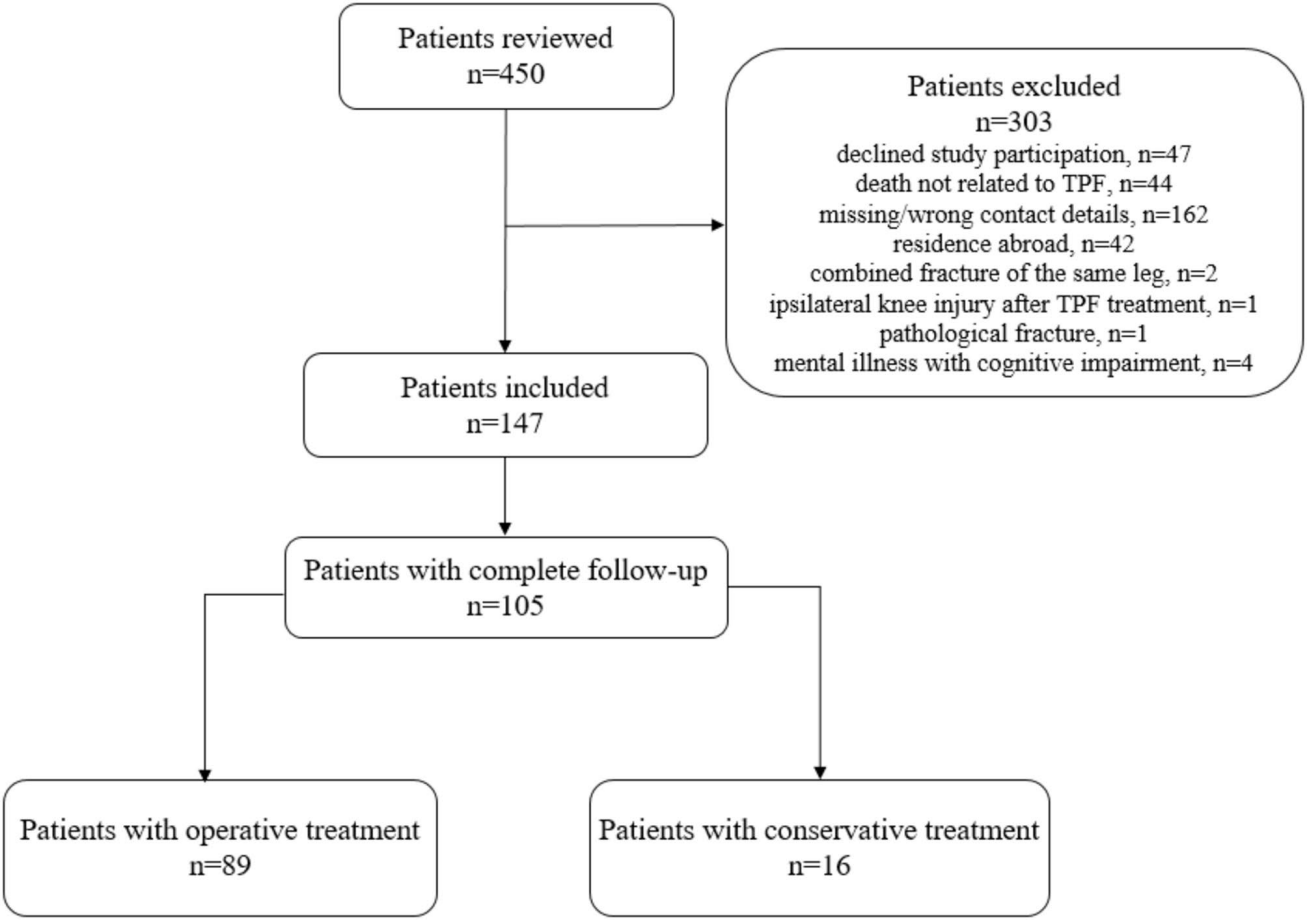
Flow diagram of included patients

After application of the exclusion criteria, 147 patients were included with 105 patients completing the follow-up (71%, Fig. 1). 89 patients underwent surgery, and 16 patients were treated conservatively. The demographic data and fracture characteristics of the study population are summarized in Table 1. The injury mechanisms are demonstrated in Table 2. Surgical treatment characteristics are presented in Table 3.

**Table 1:** Demographic data and fracture characteristics of included patients with tibial plateau fracture

	Surgical treatment			Conservative treatment		
	*n* (%)	M (SD)	Md (IQR)	*n* (%)	M (SD)	Md (IQR)
**Female sex**	50 (56)			8 (50)		
**Age** (years)		49.8 (12.5)			52.3 (17.6)	
**BMI** (kg/m²)		24.4 (3.4)			23.4 (3.4)	
**Smokers**	10 (11)			5 (31)		
**ASA risk classification**						
I	62 (70)			11 (69)		
II	24 (27)			5 (31)		
III	3 (3)			0 (0)		
**Open fracture**	1 (1)			0 (0)		
**Schatzker classification**						
I	3 (3)			5 (31)		
II	40 (45)			3 (19)		
III	18 (20)			8 (50)		
IV	11 (12)			0 (0)		
V	4 (5)			0 (0)		
VI	13 (15)			0 (0)		
**Meniscus/ligament injury**	22 (25)			2 (13)		
**Follow-up** (years)			10.5 (9–13)			10.2 (9–12)

*M* = mean; *SD* = standard deviation; *Md* = median; *IQR* = interquartile range; BMI = Body Mass Index; ASA = American Society of Anesthesiologists

**Table 2:** Injury mechanisms of included patients with tibial plateau fracture

	Surgical treatment	Conservative treatment
	*n* (%)	*n* (%)
Bicycle accident	15 (17)	1 (6)
Low-energy fall	20 (22)	6 (38)
High-energy fall	4 (5)	0 (0)
Skiing accident	27 (30)	3 (19)
Other sports accident	6 (7)	3 (19)
Motorcycle accident	7 (8)	2 (12)
Other traffic accident	10 (11)	1 (6)

**Table 3:** Performed surgical procedures

	*n* (%)	Md (IQR)
**Time trauma-definitive treatment** (days)		5 (3–9)
**External fixator**	9 (10)	
**Staged definitive treatment**	10 (11)	
**Surgical approaches**		
Anterolateral	49 (55)	
Posterolateral	2 (2)	
Posteromedial	7 (8)	
Anteromedial	5 (6)	
Combined lateral and medial	10 (11)	
Minimally invasive/percutaneous	16 (18)	
**ARIF**	18 (20)	
**Implant**		
Singular locking plate	51 (57)	
Double plate	10 (11)	
Screws	28 (32)	
**Bone graft**	34 (38)	
**Overall operation time** (minutes)		121 (80–187)
**Duration of inpatient stay** (days)		8 (6–13)

*Md* = median; *IQR* = interquartile range; ARIF = arthroscopically assisted reduction and internal fixation

### Conversion to TKA and secondary interventions

Overall, two patients (2%) required TKA, and nine patients (9%) underwent a joint-preserving procedure during the follow-up period. Both conversions to TKA occurred in the conservative treatment group (13%), 7.7 and 9.1 years following TPF. In the surgical treatment group, eight patients (9%) received a joint-preserving intervention after 4.8 years (IQR 4–7). There were four patients (5%) with intra-articular injections (all hyaluronic acid), one patient (1%) with a high tibial osteotomy for osteoarthritis of the lateral compartment, two patients (2%) with arthroscopic cartilage treatment and one patient (1%) with diagnostic arthroscopy and arthrolysis. In the conservative treatment group, one patient (6%) had an arthroscopic cartilage treatment after 10 years.

**Fig. 2:**
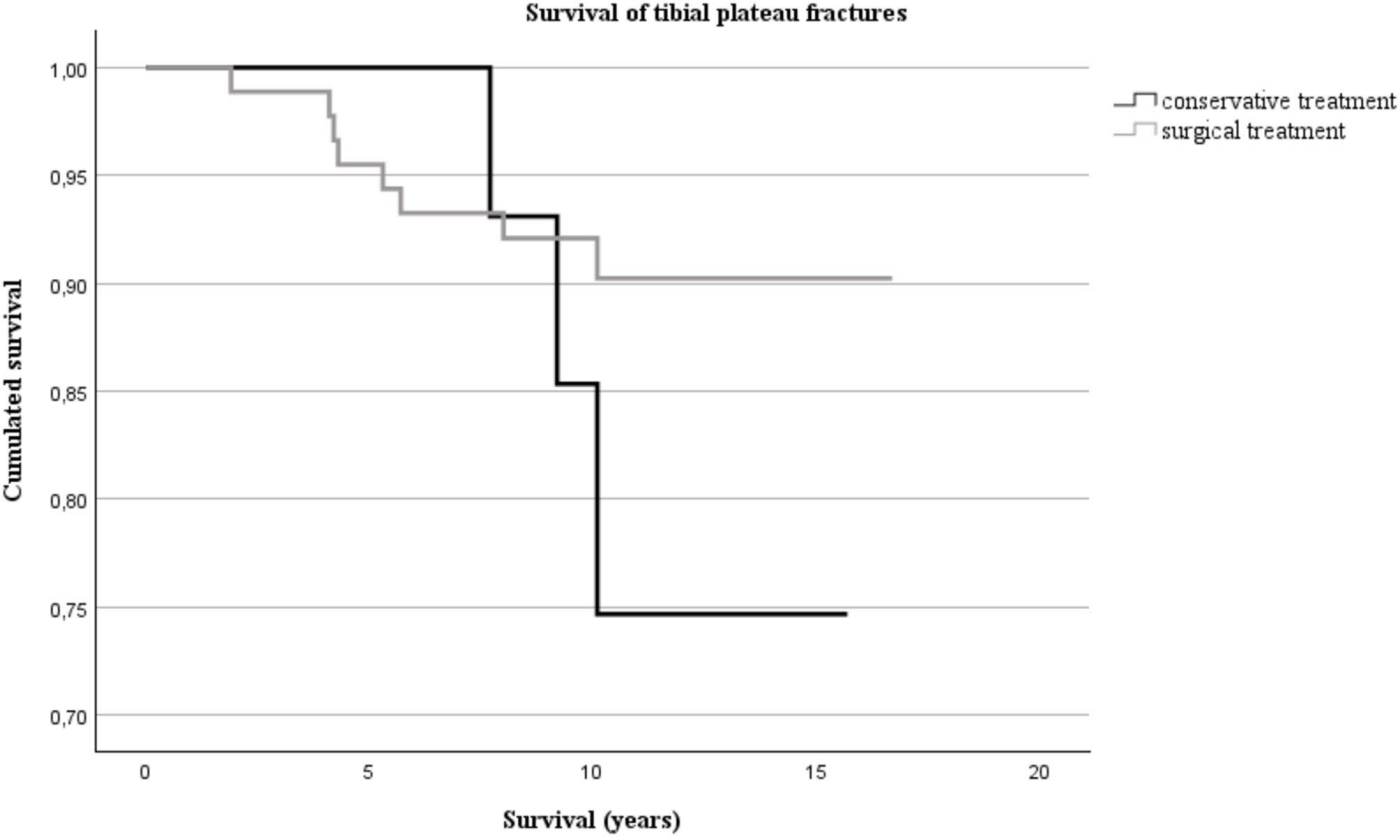
Survival curves of patients with surgical and conservative treatment of TPF and osteoarthritis-related secondary interventions as endpoint

The cumulated survival rate of surgical treatment was 96% after 5 years, 90% after 10 years and 90% after 15 years. In the conservative treatment group, the cumulated survival rate was 100% after 5 years, 75% after 10 years and 75% after 15 years (Fig. 2). Risk factors for secondary interventions after surgical treatment of TPF are depicted in Table 4. Additionally, 51 (57%) patients underwent removal of osteosynthesis material after 1.3 years (IQR 1–2).

**Table 4:** Cox proportional hazards model with hazard ratios for follow-up procedures in patients with surgical treatment of TPF

	HR	95% CI	*p*
Female sex	1.34	0.23–7.86	n.s.
Age (years)	0.97	0.90–1.10	n.s.
BMI (kg/m²)	1.40	1.12–1.74	0.03
Smokers	6.40	0.44–9.41	n.s.
ASA risk classification	0.47	0.05–4.20	n.s.
Schatzker classification	1.44	0.70-3.00	n.s.
Time trauma until surgery (days)	1.04	0.97–1.11	n.s.
Duration of surgery (minutes)	1.00	0.99–1.01	n.s.

*HR* = hazard ratio; *CI* = confidence interval; n.s.=not significant; BMI = Body Mass

Index; ASA = American Society of Anesthesiologists

### Clinical outcomes of surgical treatment

**Fig. 3:**
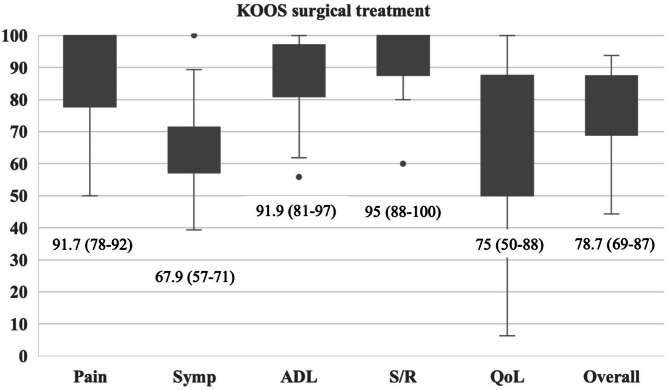
KOOS subscales “pain”, “symptoms”, “activities of daily living”, “sports and recreation”, “quality of life” and KOOS overall for surgical treatment

The overall KOOS was graded as “good” for 35 patients (39%), “fair” for 40 patients (45%) and “poor” for 14 patients (16%). The detailed results for the KOOS and its subscales are shown in Fig. 3. The mean IKDC score was 63.6 ± 16.5. The median of the TAS was 3 (IQR 3–4). A negative correlation was observed between Schatzker classification (Spearman´s r_p_ = -0.24, *p* = 0.03), duration of surgery (Spearman´s r_p_ = -0.23, *p* = 0.03), ASA risk classification (Spearman´s r_p_ = -0.28, *p* = 0.01) and the IKDC score. No further correlations were found between the variables examined and the functional scores. In the subgroup analyses, a significantly higher TAS was observed for non-smokers (median 3, IQR 3–4) compared to smokers (median 2.5, IQR 2–3, *p* = 0.02). There were no significant differences regarding the functional scores of the remaining subgroups.

A total of 17 complications (19%) were recorded during follow-up. These included two cases (2%) of superficial wound infection, two cases (2%) of deep venous thrombosis, five cases (6%) of compartment syndrome (all present at admission), one case (1%) of loss of reduction, one case (1%) of peroneal nerve injury and three cases (3%) of postoperative knee stiffness. Revision surgery was necessary in two cases (2%). Revision ORIF was performed for the patient with loss of reduction and arthroscopic arthrolysis was performed for one patient with postoperative knee stiffness.

### Clinical outcomes of Conservative treatment

**Fig. 4:**
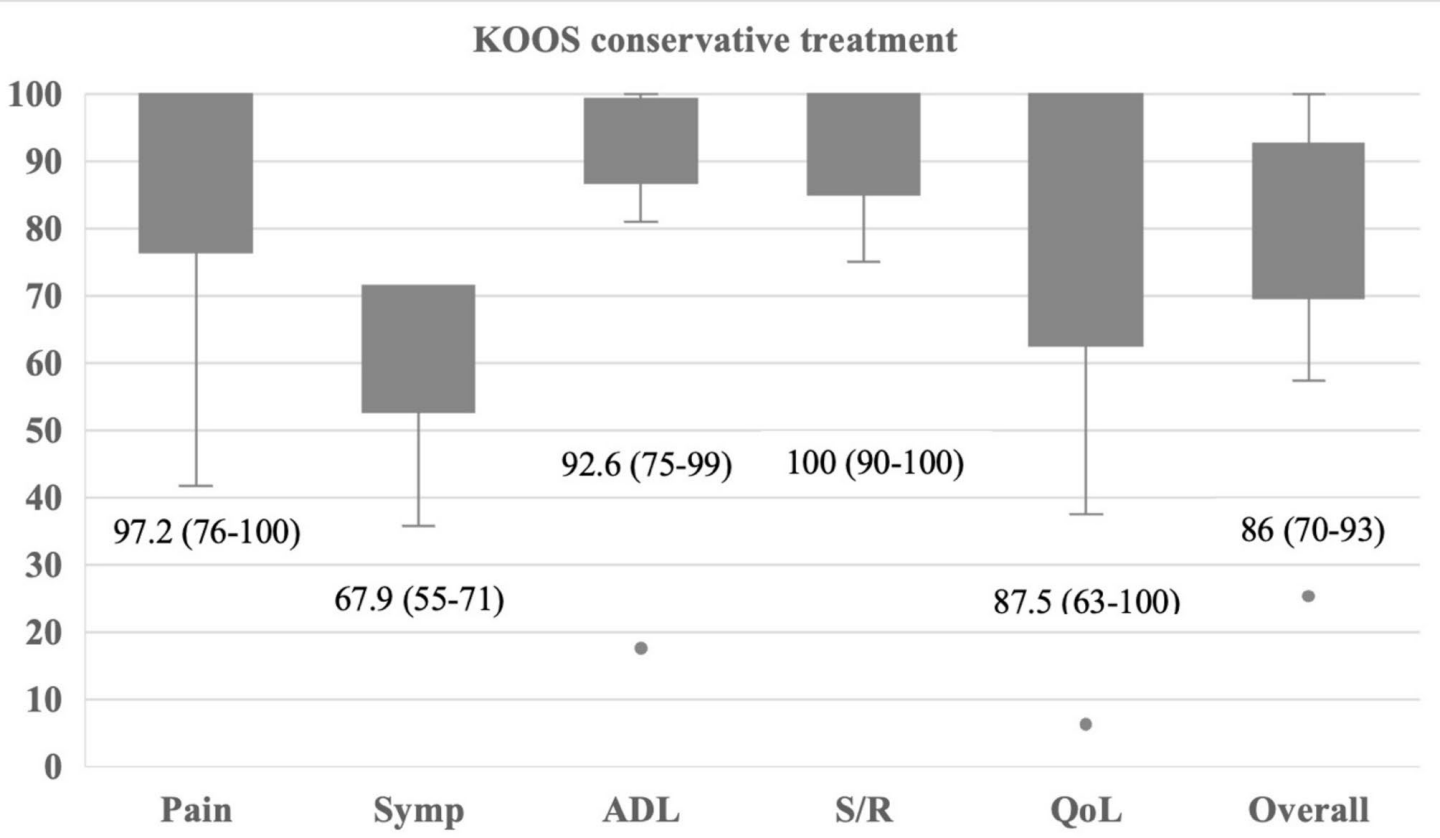
KOOS subscales “pain”, “symptoms”, “activities of daily living”, “sports and recreation”, “quality of life” and KOOS overall for conservative treatment

The overall KOOS was classified as “excellent” for one patient (8%), “good” for six patients (46%), “fair” for four patients (31%) and “poor” for two patients (15%). The exact values for the KOOS and its subscales are shown in Fig. 4. The mean IKDC score was 66.3 ± 22.2. The median TAS was 3 (IQR 3–6). A higher ASA risk classification score was associated with a lower TAS (Spearman´s r_p_ = -0.67, *p* = 0.02). No other predictive factors for functional outcomes were identified through correlation and subgroup analyses. No complications were reported during follow-up.

## Discussion

The key finding of the present study is the low incidence of osteoarthritis-related procedures following TPF with satisfactory clinical outcomes in the long-term follow-up.

Studies with mid-to-long-term follow-up demonstrated that 31–44% of patients show radiological signs of PTOA [18, 19]. Naturally, not all patients are symptomatic enough to require conversion to TKA, however, considering these numbers, the conversion rate in our cohort was markedly lower than anticipated, at just 2%. All cases that necessitated TKA over time were found in the conservative treatment group. The first case with eventual conversion to TKA involved a 60-year-old female patient with pre-existing symptomatic osteoarthritis Kellgren-Lawrence grade IV. Pre-existing osteoarthritis is a known risk factor for conversion to TKA [20]. In the second case, a 45-year-old male patient eventually required bilateral TKA, raising questions regarding the contribution of the tibial plateau fracture to the progression of osteoarthritis in this individual. A recent study of Kraml et al. found notable higher conversion rates of 6% after 2 years, 11% after 5 years and 12% after 10 years [21]. The colleagues only included surgically treated patients and almost 50% suffered from bicondylar fractures compared to 32% in the current study, which may explain the disparity in conversion rates. A systematic literature review conducted by the same research group in 2023 revealed conversion rates to TKA between 0% and 10% in the majority of studies [7].

In addition to evaluating TKA conversion rates, the present study also documented joint-preserving follow-up procedures. These procedures occurred in 9% of surgically treated patients and 6% of those treated conservatively. The most common intervention after surgical treatment of TPF was intra-articular hyaluronic acid injection, performed in 5% of patients. Scott et al. reported intra-articular injections in 14% of patients within five years after surgical treatment of TPF [11]. Although some studies have reported a significant pain-relieving effect of intra-articular hyaluronic acid injections for osteoarthritis of the knee, potentially delaying the need for prosthetic treatment, their effectiveness remains inconclusive and is subject to ongoing debate [22]. In Germany, this therapy is not routinely covered by health insurance, which may explain its lower usage in this study. Arthroscopy was performed in 3% of surgically treated patients and 6% of those treated conservatively, mostly involving cartilage therapy. Timmers et al. reported a rate of 7% for secondary arthroscopic procedures after a 7 year follow-up, though the specific types of procedures were not detailed [14]. In contrast to our findings, Mehin et al. observed joint-preserving follow-up procedures in 13% of patients with a mean follow-up of 10 years, with a threefold higher frequency in the surgical treatment group compared to the conservative treatment group [12]. In a registry-based cohort study with a mean follow-up of 14 years, Elsoe et al. identified a fivefold increased risk of secondary arthroscopy following TPF compared to a healthy control group, with secondary arthroscopies performed in 8% of cases. Male patients under the age of 50 had the highest risk within the first 5 years following TPF [13].

Due to the small sample size in the conservative treatment group, risk factors for secondary interventions could only be analysed for surgically treated patients in the present study. In contrast to the findings of Elsoe et al., no influence of age or gender was observed. Age is controversially discussed as a risk factor for subsequent conversion to TKA following TPF. For instance, Wasserstein et al. describe a 3.4% increased risk per year of age at the time of injury [8]. In a subgroup analysis of the aforementioned study by Kraml et al., patients who underwent conversion to TKA were, on average, 59 years old − 7 years older than their counterparts [21]. Kim et al., however, found no difference in the conversion rate between patients younger and older than 60 years [23]. Several studies report female gender as a risk factor for conversion to TKA [8, 10, 11, 13, 24]. This may be associated with the higher prevalence of osteoporosis in female patients and the resulting complex fracture morphologies, as complex bicondylar fractures are linked to a higher risk of PTOA and subsequent need for prosthetic treatment [8, 23, 25]. In the current study, an increased risk of follow-up interventions for patients with a higher BMI was found. Obesity is also a documented risk factor in the literature [11, 24]. The risk factors of smoking [26] and comorbidities [8, 24] which have been discussed in previous studies showed no significant association with secondary interventions in our study.

Ten years following TPF, the median KOOS of 78.7 after surgical treatment and 86 after conservative treatment can be classified as fair and good, respectively. Timmers et al. showed an overall KOOS of 66.5 seven years after surgical treatment of TPF [14]. The same study not only reports poor functional outcomes but also the highest conversion rate to TKA (22%) described in the literature. The included patients underwent surgery between 2000 and 2010, and although no details about the surgical techniques are provided, it is possible that better outcomes could be achieved with modern implants and individual therapeutic approaches by use of 360°-care for TPF. Van dreumel et al. observed a good overall KOOS of 83 with a significantly shorter follow-up than in the present study [27]. Similar to our results, Kraml et al. found a fair overall KOOS of 72.3 after nine years [21]. The IKDC score and TAS are less frequently reported in the literature on TPF. The IKDC score of 63.6 for surgical treatment and 66.3 for conservative treatment observed in this investigation is lower than the score of 82.8 reported in the study with 54 surgically treated patients with a significantly shorter follow-up of four years by Neidlein et al. [28]. We found a median TAS of 3 in both treatment groups indicating an activity level that includes light physical work, swimming, walking on uneven terrain, and forest walks. This result is slightly lower than the reported median TAS of 4 by Kraml et al. [21]. This could be attributed to both the longer follow-up and a possibly lower level of physical activity in our patient cohort, which, however, cannot be proven since we did not assess the preoperative activity level.

In the conservative treatment group, a correlation between a higher ASA score and a lower TAS was observed, while in the surgical treatment group, smokers had a lower TAS compared to non-smokers. This likely reflects an expected lower general activity level among patients with pre-existing conditions and smokers. Smoking did not show any impact on other functional scores. There are several studies that reported worse clinical outcomes in smokers. On the other hand, different investigations found no clinical relevance of smoking [29, 30]. In the surgical treatment group, correlation analysis revealed a negative impact of higher ASA risk score, greater fracture severity according to the Schatzker classification and longer surgical time on the IKDC score. Pre-existing conditions, such as diabetes, are a previously described negative predictive factor for functional outcomes after TPF [29]. Complex bicondylar fractures often result in longer operative times, as both the lateral and medial tibial plateau frequently need to be addressed through multiple surgical approaches. A correlation between longer surgical times and more complex bicondylar fracture patterns and poorer functional outcomes has been reported before [14, 21, 30]. Similar to previous studies, we could not identify any influence of the following variables on functional outcomes: gender [21, 31], age [21, 27], BMI [30, 32], low- versus high-energy trauma [31] and time between trauma and surgery [21]. However, some of these potential predictive factors are subject to controversial discussion, as some authors, in contrast to our findings, reported worse clinical outcomes for female [29], overweight [21, 29] and older [29, 32] patients. Notably, patients with and without joint-preserving follow-up interventions in the presented study showed no differences in functional outcomes. However, it remains unclear whether the results in this cohort would have been significantly worse without the secondary procedures or if the interventions were ineffective, as we were unable to analyse scores prior to the follow-up interventions.

This study has some limitations. The retrospective nature of the study carries the risk of recall bias. Despite multiple contact attempts, only 105 of 147 included patients (71%) completed the follow-up. This poses the risk of selection bias, for instance, if dissatisfied patients were less likely to participate in the study. It is an issue that other studies with similarly long follow-up periods have also reported [21, 29]. The functional outcomes were assessed using scores, and no clinical follow-up of the patients was conducted. Furthermore, there was no evaluation of radiological variables such as the exact amount of initial fracture displacement or articular depression, postoperative reduction quality, mechanical limb alignment and radiological singns of PTOA, as these factors are sufficiently investigated in the literature [32–34]. Due to the long follow-up it was impossible to account for all variables that could impact the outcome measures such as other conditions and patient factors. Thus, the factors identified as correlated with our outcomes should not be regarded as the sole influences on outcome after TPF. The sample size of conservatively treated patients was insufficient and the groups were too heterogeneous to conduct a meaningful comparison between the outcomes of conservative and surgical treatment for undisplaced or minimally displaced fractures.

Nevertheless, this is one of the largest studies with a median follow-up of more than ten years investigating the rates of TKA and other secondary procedures after TPF as well as the long-term clinical outcomes with corresponding predictive factors. All patients were treated at a single level I university trauma centre, which ensured good comparability of the patients.

## Conclusions

Despite the severity of TPF, which carry a high risk of PTOA, the present study found a low incidence of TKA and other joint-preserving, osteoarthritis-related procedures. With appropriate indications, satisfactory long-term clinical outcomes can be achieved with both conservative and surgical treatments. Patients with a high BMI should be treated with special care because of their increased risk for secondary interventions. Additionally, the expectations of patients with a higher ASA risk score and complex fractures, accompanied by longer surgical times, should be managed carefully to ensure a realistic outlook on functional outcomes.

## Data Availability

The data that support the findings of this study are available from the corresponding author upon request.

## Abbreviations

ARIF: Arthroscopically assisted reduction and internal fixation
ASA: American Society of Anesthesiologists
BMI: Body mass index
CI: Confidence interval
CT: Computed tomography
e.g.: For example
HR: Hazard ratio
IKDC: International Knee Documentation Committee
IQR: Interquartile range
KOOS: Knee injury and Osteoarthritis Outcome Score
M: Mean
MRI: Magnetic resonance imaging
NPWT: Negative pressure wound therapy
n.s.: Not significant
ORIF: Open reduction and internal fixation
PTOA: Post-traumatic osteoarthritis
R: Range
SD: Standard deviation
TAS: Tegner Activity Score
TKA: Total knee arthroplasty
TPF: Tibial plateau fracture
